# Epigenetic Changes of *CXCR4* and Its Ligand *CXCL12* as Prognostic Factors for Sporadic Breast Cancer

**DOI:** 10.1371/journal.pone.0029461

**Published:** 2011-12-29

**Authors:** Edneia A. S. Ramos, Mariana Grochoski, Karin Braun-Prado, Gerusa G. Seniski, Iglenir J. Cavalli, Enilze M. S. F. Ribeiro, Anamaria A. Camargo, Fabrício F. Costa, Giseli Klassen

**Affiliations:** 1 Epigenetic and Molecular Biology Laboratory, Department of Basic Pathology, Federal University of Parana, Parana, Brazil; 2 Cancer Biology and Epigenomics Program, Children's Memorial Research Center, Feinberg School of Medicine, Northwestern University, Chicago, Illinios, United States of America; 3 Department of Genetics, Federal University of Parana, Parana, Brazil; 4 Laboratory of Molecular Biology and Genomics, Ludwig Institute for Cancer Research, São Paulo, São Paulo, Brazil; Health Canada, Canada

## Abstract

Chemokines and their receptors are involved in the development and cancer progression. The chemokine CXCL12 interacts with its receptor, CXCR4, to promote cellular adhesion, survival, proliferation and migration. The *CXCR4* gene is upregulated in several types of cancers, including skin, lung, pancreas, brain and breast tumors. In pancreatic cancer and melanoma, *CXCR4* expression is regulated by DNA methylation within its promoter region. In this study we examined the role of cytosine methylation in the regulation of *CXCR4* expression in breast cancer cell lines and also correlated the methylation pattern with the clinicopathological aspects of sixty-nine primary breast tumors from a cohort of Brazilian women. RT-PCR showed that the PMC-42, MCF7 and MDA-MB-436 breast tumor cell lines expressed high levels of *CXCR4*. Conversely, the MDA-MB-435 cell line only expressed *CXCR4* after treatment with 5-Aza-CdR, which suggests that *CXCR4* expression is regulated by DNA methylation. To confirm this hypothesis, a 184 bp fragment of the *CXCR4* gene promoter region was cloned after sodium bisulfite DNA treatment. Sequencing data showed that cell lines that expressed *CXCR4* had only 15% of methylated CpG dinucleotides, while the cell line that not have *CXCR4* expression, had a high density of methylation (91%). Loss of DNA methylation in the *CXCR4* promoter was detected in 67% of the breast cancer analyzed. The absence of *CXCR4* methylation was associated with the tumor stage, size, histological grade, lymph node status, *ESR1* methylation and *CXCL12* methylation, metastasis and patient death. Kaplan-Meier curves demonstrated that patients with an unmethylated *CXCR4* promoter had a poorer overall survival and disease-free survival. Furthermore, patients with both *CXCL12* methylation and unmethylated *CXCR4* had a shorter overall survival and disease-free survival. These findings suggest that the DNA methylation status of both *CXCR4* and *CXCL12* genes could be used as a biomarker for prognosis in breast cancer.

## Introduction

Breast cancer is a major public health issue worldwide. In 2004, the most recent year available for global data, there were 1.15 million new breast cancer cases and over 500,000 deaths reported worldwide [1]. Although advances have been made in reducing the mortality rates and improving survival, cancer is still the leading cause of death among men and women under 85 years of age in the United States [2]. In Brazil, 49,420 new cases of breast cancer have been estimated to occur between 2010 and 2011 [3]. Data from the Unique System of Heath (SUS) demonstrated that the mortality rates for breast cancer are 12.6 out of every 100,000 cases in Brazilian women (http://mortalidade.inca.gov.br). Metastases cause 90% of human cancer deaths [4]. For breast cancer, due to the inability to accurately predict the risk of metastasis, more than 80% of patients receive adjuvant chemotherapy. However, approximately 40% of these patients still relapse and die of metastatic breast cancer within five years [4].

Generally, cancer is described as a disease driven by progressive genetic abnormalities involving mutations in oncogenes and tumor suppressor genes as well as other chromosomal aberrations [5]. Breast cancer, similar to other types of cancer, is driven by epigenetic alterations, which do not affect the primary DNA sequence [6], [7], [8]. These alterations lead to aberrant transcriptional regulation, which results in changes in the expression pattern of genes implicated in many cellular functions. These epigenetic alterations include changes in DNA methylation and histone modifications [7]. DNA hypermethylation is frequently associated with gene repression and genomic instability through silencing of the DNA repair genes, and several genes have been shown to be silenced in different steps of breast cancer [9], [10].

Although the list of hypermethylated genes involved in the tumorigenesis of breast cancer has increased, much of the focus has remained on the estrogen receptor alpha (*ESR1*) and progesterone receptor (*PGR*) as these proteins have been implicated in breast cancer development and progression [7]. These genes are viable prognostic markers, and approximately 70% of patients are suitable candidates for endocrine therapy [11]. The HER2 protein, which is present in approximately 30% of patients, serves as another important molecular prognosis marker for breast cancer and makes tumors suitable for herceptin antibody treatment [12]. However, despite the existence of well-documented molecular markers, breast cancer deaths remain a major public health issue.

Understanding the molecular mechanisms involved in breast cancer initiation and progression could provide strategies to identify new diagnostic and prognostic markers as well as better treatment for the disease. Thus, we evaluated the expression pattern and methylation status of the *CXCR4* gene, which encodes a well-known protein involved in breast cancer. The CXCR4 chemokine together with its ligand, CXCL12, are involved in the mechanism of breast cancer metastasis. Breast cancer cells from primary tumors over-expressing CXCR4 are attracted to CXCL12 expressing cells in the lung, lymph nodes, liver or bones, which leads to the metastasis of detached tumor cells [13]. Immunohistochemical analyses have shown that specific patterns of CXCR4 expression (i.e., in the nucleus or cytoplasm) are correlated with a high nuclear grade [14] or lymph node metastasis [15], [16]. Recent studies have indicated that the epigenetic mechanisms that negatively regulate the expression of *CXCL12* and *ESR1* are involved in breast cancer metastasis and correlate with poor survival of patients [17]. Additionally, in melanoma and pancreatic cancer, the *CXCR4* promoter is regulated by increased DNA methylation, which results in lower *CXCR4* mRNA expression [18], [19].

In this study, we evaluated the methylation pattern of the *CXCR4* gene promoter in breast tumor cell lines and primary tumor samples and correlated this pattern with clinicopathological data. We also compared the results from the *CXCR4* DNA methylation study with the results from our previous *CXCL12* study [17]. Together, these results suggest that the epigenetic regulation by DNA methylation of both the *CXCR4* and *CXCL12* genes in breast cancer could serve as a potential biomarker to indicate patient prognosis.

## Results

### *CXCR4* expression in breast tumor cell lines

**Figure 1:**
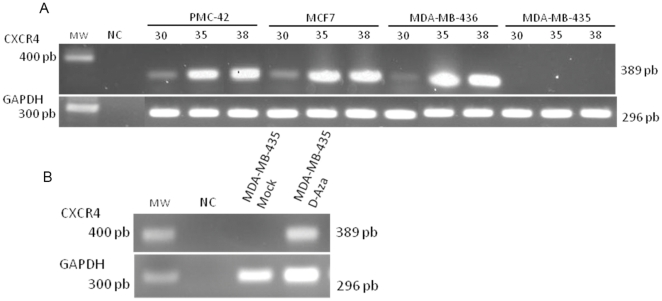
*CXCR4* expression analysis using semi-quantitative RT-PCR in breast cancer tumor cell lines and *CXCR4* expression after 5-aza-2′-deoxycytidine (D-Aza) treatment. (A) The bands represent *CXCR4* expression in the PMC-42, MCF7, MDA-MB-436 cell lines and (B) MDA-MB-435 mock or MDA-MB-435 D-Aza represent the MDA-MB-435 cell line before and after treatment with 5-aza-2′-deoxycytidine, respectively. The *GAPDH* gene was used as a positive control in both experiments. MW, Molecular Weight, NC represents the PCR reaction without DNA (negative control).

The expression pattern of *CXCR4* in four breast tumor cell lines was evaluated using RT-PCR. A 389 bp transcript corresponding to the *CXCR4* gene was detected in the PMC-42, MCF-7 and MDA-MB-436 cell lines (Fig. 1A). In contrast, *CXCR4* expression was not detected in the MDA-MB-435 cell line. To determine if *CXCR4* expression was lost, all analyses were repeated at least twice. *GAPDH* expression was detected in all samples tested (Fig. 1A).

To confirm the epigenetic transcriptional silencing of *CXCR4* in breast cancer, we treated the MDA-MB-435 cell line with the demethylating agent 5-aza-2′-deoxycytidine (5-aza-CdR). As previously demonstrated in pancreatic and melanoma cell lines, the expression of *CXCR4* was restored in the MDA-MB-435 cells upon 5-aza-CdR treatment (Fig. 1B) [18], [19].

### *CXCR4* silencing by DNA methylation

Sato et al. (2005) [18] analyzed four DNA areas within the 5′ upstream region of the *CXCR4* gene using a combined bisulfite restriction analysis (COBRA) method. Their work demonstrated that *CXCR4* is regulated by DNA methylation in human pancreatic cancer cell lines within the TSS region, which contains the majority of the methylated CpG dinucleotides. In our work, we therefore selected the TSS region, comprised of nucleotides from the positions −173 to +11 in the *CXCR4* promoter, to analyze in our breast tumor cell lines and tumor samples.

**Figure 2:**
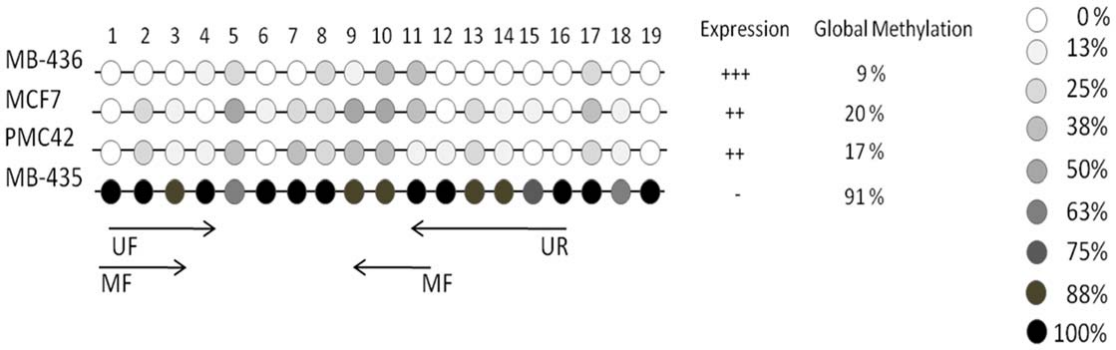
Bisulfite sequencing of the *CXCR4* gene promoter in the breast cancer cell lines. The cell lines used are shown. The nineteen dinucleotides are numbered in agreement with the sequence. The open circles represent the unmethylated dinucleotides while the gray to black portion represents the percentage of methylation. On the right side methylation pattern are represented according to data of RT-PCR and the absolute percentage value. The arrows below the CpG dinucleotides represent the MSP primers that were used.

Sodium bisulfite sequencing was performed on 184 bp DNA fragment containing 19 CpG dinucleotides. The methylation patterns of eight independent *CXCR4* alleles in the PMC-42, MCF7, MDA-MB-436 and MDA-MB-435 cell lines were analyzed. The *CXCR4*-negative cell line, MDA-MB-435, demonstrated a hypermethylation of 91% of the CpG dinucleotides (Fig. 2). The high density of cytosine methylation explains the inactivation of *CXCR4* in the MDA-MB-435 cell line, which was demonstrated by RT-PCR (Fig. 1A). This inactivation due to hypermethylation was confirmed by treatment with 5-aza-2′-deoxycytidine, a demethylating agent, which resulted in the subsequent expression of *CXCR4* (Fig. 1B). In contrast, the cell lines that expressed *CXCR4*, which were PMC-42, MCF-7 and MDA-MB-436, had lower levels of CpG dinucleotide methylation (i.e., 17%, 20% and 9%, respectively) (Fig. 2). The presence of a greater number of unmethylated CpG dinucleotides may explain the expression of the *CXCR4* gene in these cell lines, which was verified by RT-PCR (Fig. 1A).

The methylation profile comparing the *CXCR4*-expressing cell lines (MCF7, PMC-42 and MDA-MB-436) to the non-expressing cell line (MDA-MB-435) demonstrated that the differentially methylated dinucleotides were CpGs 1–4 and 9–16 (Fig. 2). Since these differentially methylated CpGs may regulate the silencing of the *CXCR4* gene, these regions were subsequently analyzed in primary tumors samples using Methylation-Specific PCR (MSP).

### MSP analysis in breast tumor cell lines

**Figure 3:**
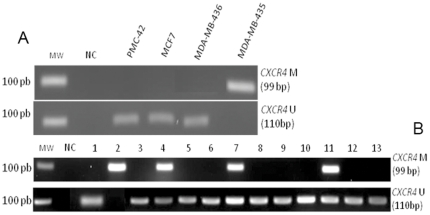
MSP analysis in breast cancer cell lines and primary breast tumors. (A) Primer standardization for methylated and unmethylated conditions in tumor cell lines. (B) MSP analysis of primary tumors. Thirteen samples are represented. MW, Molecular Weight; NC, Negative Control.

CpG dinucleotides 1 to 4 and 9 to 16, which lie within a region that is differentially methylated, were chosen for MSP analysis (as described in material and methods) (Fig. 2). The MSP technique was tested with DNA from the tumor cell lines to confirm if this DNA region could be used to analyze the *CXCR4* methylation pattern in primary tumors (Fig. 3A). RT-PCR results from the cell lines were then used to compare the pattern of gene expression to the presence or absence of DNA methylation detected by the MSP technique. The MDA-MB-435 breast tumor cell line showed a methylated fragment in the *CXCR4* CpG island, which correlated with the lack of *CXCR4* expression in this cell line. In contrast, the PMC-42, MCF7 and MDA-MB-436 cell lines, which express *CXCR4*, demonstrated only unmethylated fragments (Fig. 3A). Therefore, the MSP results from the breast tumor cell lines corroborated with both the RT-PCR and sequencing data.

### MSP analysis in primary breast tumors

The MSP assay was subsequently used to analyze the methylation of the *CXCR4* gene in primary breast tumor samples. For the methylated and unmethylated conditions, thirteen representative tumor samples are shown (Fig. 3B). From all the samples tested (69), only three contained both methylated and unmethylated CpG dinucleotides. Based on this result, we concluded that *CXCR4* could be partially silenced, or the mechanism of silencing could progress during the tumorigenesis process (Fig. 3B). However, a lack of methylation of the *CXCR4* gene was found in the majority of the samples with 46 out of the 69 samples (67%) not showing CpG methylation in the region evaluated by MSP.

### Correlations between the *CXCR4* promoter methylation status and clinicopathological data

Sato *et al.* (2005) [18] analyzed a DNA region comprised of nucleotides from the −173 to +11 positions in the *CXCR4* promoter. This region was shown to contain 19 CpGs, which were used to determine the methylation pattern of the *CXCR4* gene and correlates this pattern with *CXCR4* gene silencing in human pancreatic cancer cell lines. In this study, we used this region to correlate the CpG island methylation pattern of the *CXCR4* gene with the clinical and pathological parameters shown in Table 1. Unmethylated *CXCR4* was not significantly associated with the age of disease onset (*p* = 0.466), estrogen receptor status (*p* = 0.310), HER2 expression (*p* = 0.276), progesterone receptor status (*p* = 0.117), tumor recurrence (*p* = 1.000) or histological type (*p* = 0.849). However, unmethylated *CXCR4* did correlate with the tumor stage (*p*<0.001), tumor size (*p*<0.001), histological grade (*p*<0.001), SBR grade (*p*<0.001), lymph node status (*p* = 0.002), metastasis (*p* = 0.026) and death (*p* = 0.038). We also found that the majority of the samples with more advanced stages (II or III/IV) were unmethylated (82% and 94%, respectively). Similar results were observed for the tumor size (pT3/T4 = 95%) and SBR grade (III = 95%). Additionally, samples that were positive for lymph nodes had a higher percentage of unmethylated *CXCR4* (82%). We also correlated the methylation pattern of the *CXCR4* gene promoter with the methylation status of the *ESR1* and *CXCL12* genes, which had been previously studied by our group [17]. Unmethylated *CXCR4* was significantly associated with methylated *ESR1* and *CXCL12* (*p* = 0.006 and *p* = 0.001, respectively).

**Table 1:** Clinicopathological features of 69 patients with primary breast carcinomas and methylation status of *CXCR4* gene.

Variables	Samples (%)	*CXCR4* Methylation		*p value*
		Yes (%)	No (%)	
**Age**				
<45	9 (13)	2 (22)	7 (78)	0.466
≥45	60 (87)	24 (40)	36 (60)	
**Stage**				
I	19 (27)	14 (74)	5 (26)	**<0.001**
II	33 (48)	6 (18)	27 (82)	
III/IV	17 (25)	1 (6)	16 (94)	
**Tumour size**				
pT1	17 (25)	12 (71)	5 (29)	**<0.001**
pT2	35 (50)	7 (20)	28 (80)	
pT3/pT4	17 (25)	3 (18)	14 (82)	
**SBR**				
I	19 (28)	14 (74)	5 (26)	**<0.001**
II	32 (46)	7 (22)	25 (78)	
III	18 (26)	1 (5)	17 (95)	
**Lymph node status**				
Positive	33 (48)	6 (18)	27 (82)	**0.002**
Negative	35 (52)	19 (42)	16 (58)	
**Estrogen receptor (RE)**				
Positive	57 (84)	21 (37)	36 (63)	0.310
Negative	11 (16)	2 (18)	9 (82)	
**HER-2**				
Positive	19 (31)	4 (21)	15 (79)	0.276
Negative	43 (69)	15 (35)	28 (65)	
**Progesterone receptor (PR)**				
Positive	46 (74)	16 (35)	30 (65)	0.117
Negative	16 (26)	2 (12)	14 (88)	
***ESR1*** ** Methylation** *				
M	28 (41)	6 (21)	22 (79)	**0.006**
U	40 (59)	22 (55)	18 (45)	
***CXCL12*** ** Methylation** *				
M	37 (54)	12 (32)	25 (73)	**0.001**
U	32 (46)	21 (67)	11 (33)	
**Metastasis**				
Positive	21 (30)	3 (14)	18 (86)	**0.026**
Negative	48 (70)	20 (42)	28 (58)	
**Death**				
Positive	17 (25)	3 (18)	14 (82)	**0.038**
Negative	50 (75)	23 (46)	27 (54)	
**Recurrence**				
Positive	10 (14)	3 (30)	7 (70)	1.000
Negative	59 (86)	20 (34)	39 (66)	
**Histogical type**				
Ductal Carcinoma Invasive	50 (72)	17 (34)	33 (66)	0.849
Lobular Carcinoma Invasive	19 (28)	6 (32)	13 (68)	

**Abbreviations:** *p*, value from statistical analysis *χ^2^* test and Fisher's exact test; M, methylated; U unmethylated; significant data are in bold.

*CXCL12 and *ESR1* methylation data were used from a previous study published by our group [17].

**Figure 4:**
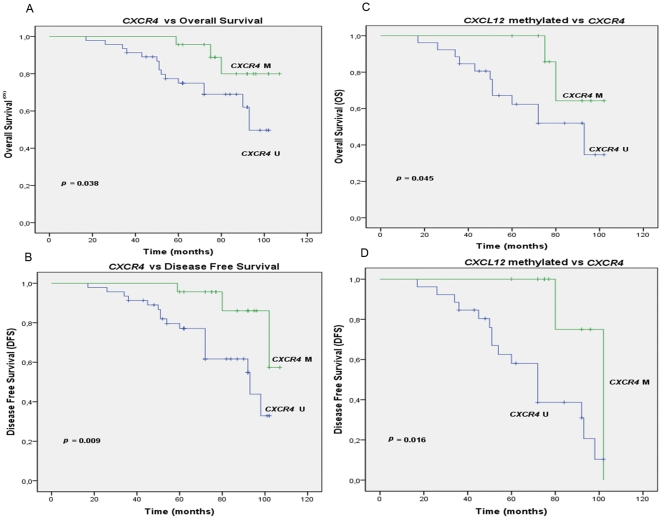
Kaplan Meier curves for overall survival and disease-free survival according to the methylation status of *CXCR4* and *CXCL12*. *CXCR4* methylation status and the correlation with (A) overall survival (OS) and (B) disease-free survival (DFS) are shown. *CXCL12* methylation status and association to *CXCR4* methylation for (C) OS and (D) DFS are shown.

These results suggest that the CpG island methylation in the *CXCR4* gene may be an important prognostic factor for breast cancer. To test this hypothesis, we analyzed all of the clinical and clinicopathological data for prognostic value in a univariate analysis for disease-free survival (DFS) and overall survival (OS) using Kaplan-Meier Curves (*p* values were generated using the log rank test). The tumors from patients with an unmethylated *CXCR4* gene had a significantly poorer prognosis than the patients with tumors containing CXCR4 methylation for overall survival (OS) (*p* = 0.038) and disease-free survival (DFS) (*p* = 0.009) (Fig. 4A and B). To identify the impact of signaling between *CXCL12* and *CXCR4*, we generated the Kaplan-Meier curves with a combination of both genes. These data demonstrated that patients with hypermethylated *CXCL12* and unmethylated *CXCR4* had a shorter OS and DFS (*p* = 0.045 and *p* = 0.016, respectively) (Fig. 4C and D). Thus, methylated *CXCL12*, which was previously studied as a marker of poor patient prognosis [17], may be accompanied by a process involving the unmethylated or hypomethylated *CXCR4* gene promoter. While the mechanism behind these processes remains to be identified, these results agree with the hypothesis that tumoral cell dissemination may occur with the silencing or absence of CXCL12 in the same cells over-expressing the CXCR4 protein.

## Discussion

Breast cancer is the most common malignant tumor affecting women worldwide. Metastasis is an important feature of malignant tumors and has a major impact on the prognosis and therapeutic decisions for patients. The metastatic process is multi-factorial, non-random and exhibits organ selectivity. Lymph node metastases are the most frequently occurring type of metastatic lesion [20]. Chemokine receptors are defined by their ability to induce the directional migration of cells toward a chemotactic cytokine gradient, and the CXCR4 receptor is essential for development, hematopoiesis, organogenesis and vascularization [21]. Muller *et al.* demonstrated that CXCR4 is undetectable in normal mammary gland tissue but is consistently expressed in human breast cancer cells and metastases. The ligand of CXCR4, CXCL12, is preferentially expressed in organs targeted by metastases, such as the lungs, liver, bone marrow and lymph nodes [13]. Additional reports have demonstrated that a high CXCR4 expression pattern correlates with lymph node metastases from invasive ductal breast cancer [15], [16].

Recent emphasis has been placed on the critical role of epigenetic changes, especially DNA methylation and histone modifications, in human carcinogenesis. Epigenetic changes differ from genetic changes as they occur at a higher frequency, are reversible upon treatment with pharmacological agents and occur at defined regions within genes [7]. The *CXCR4* gene has been shown to be epigenetically regulated in endometrial carcinoma [22], melanoma [19], colonic carcinoma [23] and pancreatic cancer [18]. In this study, we investigated the methylation status of the 5′ TSS region of the *CXCR4* promoter in primary breast tumor samples.

First, we evaluated the regulation of the *CXCR4* gene by DNA methylation in breast cancer cell lines. MDA-MB-435 demonstrated a repression of *CXCR4* gene expression, which was restored after 5-aza-CdR treatment (Fig. 1A and 1B). This result agrees with the data from pancreatic cancer cell lines [18]. Then, a 184 bp fragment of the CXCR4 promoter, which harbored the TSS motif that contains 19 CpGs, was sequenced in breast cancer cell lines. This region was differentially methylated according to the *CXCR4* expression levels (Fig. 2). The MSP technique was then used to demonstrate that 46 of the 69 samples analyzed were not methylated (67%). These data are novel for breast cancer since only pancreatic primary carcinomas have previously been studied for *CXCR4* DNA methylation. In pancreatic cancer, *CXCR4* methylation occurred in 46% of the tumors but did not display any significant associations with common clinicopathological factors, such as age, gender, stage or lymph node metastasis [18].

The importance of oncogene methylation in cancer is still poorly understood. The inactivation of oncogenes confers a selective disadvantage to tumor cells by threatening the survival of the cell and negatively affecting carcinogenesis [24]. Muller *et al.* [13] showed that *CXCR4* gene expression is absent or down regulated in normal breast cells, and this result was also confirmed in other tumor cell types. Singh *et al.* (2004) observed that the *CXCR4* mRNA and protein levels were significantly higher in prostate cancer cell lines (PC3 and LNCaP) compared to normal prostate epithelial cells (PrEC) [25]. A similar finding was reported by Meier *et al.* (2007) in neuroblastoma cell lines where invasive cells lines (IGR-N91, SH-SY5Y) had high expression levels of *CXCR4*, whereas a non-invasive neuroblastoma cell line (IGR NB8) expressed low levels of the *CXCR4* gene [26]. These data suggest that mechanisms, likely including DNA methylation, exist in normal cells to reduce the expression of *CXCR4*. Thus, cancer progression could lead to the demethylation of the *CXCR4* promoter to selectively favor tumor growth and cell migration.

Tumors with a poor prognosis in our study, such as stage III (94%), tumor size T3/T4 (82%) or SBR III (95%), had unmethylated *CXCR4* (*p*<0.001). The demethylation of an oncogene, such as *CXCR4*, could be involved in processes such as cell migration and metastasis. Thus, the regulation of this gene deserves attention for its involvement in disease progression. Recent work by Hiller *et al.* showed an association between *CXCR4* over-expression and patient outcome [27]. This group analyzed the association of locally advanced breast cancer (stages IIB or III of the TNM staging system) and *CXCR4* expression after neoadjuvant therapy. The survival was poor for patients whose *CXCR4* expression levels remained high following neoadjuvant therapy [27].

Our study showed a statistical correlation between a positive lymph node status and unmethylated *CXCR4* (*p* = 0.002). A similar correlation was observed between unmethylated *CXCR4* and the presence of metastases (86% of the samples were unmethylated) (*p* = 0.026) and non-survival (82% of the samples were unmethylated) (*p* = 0.038). These results showed a correlation between a poor prognosis and an unmethylated *CXCR4* promoter. Data discussing the expression of *CXCR4* and the association of the CXCR4 with metastasis in the literature are somewhat controversial. Andre *et al.* (2006) found a correlation between CXCR4 expression and liver metastases but no correlation was found between the expression of the CXCR4 protein with various clinicopathological variables, such as age, tumor grade, estrogen receptor status or HER2 expression [28]. Kang *et al.* (2005) reported an association between high CXCR4 protein levels and lymph node metastasis but not with distant metastases [29]. Kato *et al.* (2003) examined the CXCR4 staining patterns in focal and diffuse-type tumors and found no significant differences in the pathological types, histological grades or estrogen receptor statuses of the tumor types [14]. However, a significant correlation was observed between the CXCR4 protein level and the degree of lymphatic spread but not hematogenous metastases [15]. Holm *et al.* (2007), opposed to Andre *et al.* (2006) [28], found a significant correlation between a high CXCR4 protein expression level and a HER2-negative status [30]. Conversely, Woo *et al.* (2008) found a significant association between a high nuclear expression of CXCR4 with the occurrence of metastasis in the lymph nodes. According to this study, tumors that were CXCR4+/lymph node+ were associated with a negative ER and PR status [31]. Kang *et al.* (2005) found no correlation between CXCR4 expression and overall survival or disease-free survival of patients but found statistically significant higher levels of CXCR4 protein in node-positive tumors [29]. The expression of CXCR4 was also higher among patients with distant metastases, but no significant correlation between these factors was found [29].

The lack of correlation between CXCR4 protein expression and a positive lymph node status or distant metastases was discussed by Shim *et al.* (2006) [32]. They observed a high expression of CXCR4 in primary tumors, whereas cytoplasmic expression of this receptor was undetected in most secondary lymph nodes tumors. The reduced expression of CXCR4 on the cell surface can be justified by the high expression of the CXCL12 protein in the lymph nodes [32] as CXCL12 stimulates the internalization and subsequent lysosomal degradation of CXCR4.

Furthermore, we evaluated the correlation between unmethylated *CXCR4* and the hypermethylation of other genes strongly associated with breast cancer. Our previous results with the same patients showed that the methylation of *ESR1* and *CXCL12* occurred at higher frequencies in patients with metastases and death [17]. In this study, we evaluated whether unmethylated *CXCR4* with concurrent *CXCL12* hypermethylation produced a more aggressive disease phenotype. Tumors from patients with an unmethylated *CXCR4* gene had a significantly poorer OS and DFS compared to patients with tumors containing methylated *CXCR4* (*p* = 0.038 and *p* = 0.009, respectively) (Fig. 4A and B). However, Kaplan-Meier analysis demonstrated that patients with unmethylated *CXCR4* and methylated *CXCL12* had shorter overall and disease free survivals (*p* = 0.045 and *p* = 0.016, respectively) (Fig. 4C and D). The molecular mechanisms facilitated by *CXCL12* and its partner, *CXCR4*, which result in the poor prognosis for these patients remain obscure.

Previously, with the same patient cohort, we demonstrated that *ESR1* was inactivated by DNA hypermethylation, which resulted in the loss of the receptor for the estrogen protein (ER) [17]. We hypothesized that the decrease of estrogen would lead to all ER target genes becoming susceptible to epigenetic silencing [33], including *CXCL12* [17] and the unmethylated *CXCR4* promoter, which would thus lead to a more aggressive disease.

The main limitation of our study was the small sample size. However, even with the limited number of samples, we were able to observe strong correlations between epigenetic changes in both *CXCR4* and *CXCL12* and a poor prognosis. We believe that the statistical differences found here underline the importance of changes in the DNA methylation of chemokines and their receptors in the process of tumor progression. These new discoveries may provide a molecular prognostic factor for breast cancer and may help to develop therapies that are more effective for this type of cancer. Our results could also open new avenues for a more efficient management of metastatic disease in breast cancers.

In summary our data demonstrate for the first time that *CXCR4* gene expression in primary breast tumors is regulated by DNA methylation, and *CXCR4* methylation associates with several clinicopathological parameters. Loss of DNA methylation in the promoter region of *CXCR4* correlated with a more aggressive disease in terms of tumor stage, tumor size, SBR grade, 5demonstrated that concurrent epigenetic changes of *CXCR4* and its ligand, *CXCL12*, correlated with shorter disease-free and overall survivals. We believe that our findings will be important for a better understanding of metastatic disease; however, more research is needed to unveil additional molecular mechanisms associated with the metastatic process.

## Materials and Methods

### Cell lines

Breast tumor cell lines were obtained from the Ludwig Institute for Cancer Research (São Paulo, Brazil). The following cell lines were used: MDA-MB-436, MDA-MB-435, MCF7 and PMC-42. The cell lines were cultured at 37°C in a humidified incubator with 5% CO_2_ in RPMI 1640 medium containing 10% fetal bovine serum supplemented with 0.2 mM glutamine and 40 µg/mL gentamicin.

### Patient samples

For the methylation analysis, frozen samples of breast tumors (n = 69) were obtained from breast cancer patients treated by primary surgery at the Nossa Senhora das Graças Hospital, Curitiba, PR, Brazil, with institutional approval. The study included female patients with invasive breast tumors. All patients gave informed consent for their tissue to be retained and analyzed for research purposes. The ages of the patients ranged from 27 to 84 years (mean 57.8±14.7). The histological types of the tumors were either infiltrative ductal carcinoma (IDC) (n = 51, 74%) or infiltrative lobular carcinoma (ILC) (n = 18, 26%). The lymph node statuses of the patients were determined and included 51% positive (n = 35) and 49% negative (n = 33) samples. The histological grades of the tumors were determined according to the modified Bloom-Richardson criteria. Of the patients analyzed, 28% were Grade I, 48% were Grade II and 24% were Grade III. TNM staging was determined according to the World Health Organization (WHO) classification [34]. The tumor samples were the same samples used by Ramos *et al.* (2010) [17]. The patients' clinicopathological data are shown in Table 1.

#### Ethics Statement

All patients gave their informed written consent for their tissues to be retained and analyzed for research purposes. All signed consent forms are in the custody of the corresponding author. This study was approved by the National Committee of Ethics in Research with the process number 25000.007020/2003-93. Institutional approval was granted by the Ethics Committee of Human Beings Research from the Federal University of Parana (UFPR) with the register number 7220-251/2003 (20/02/2003).

### Immunohistochemistry

Immunohistochemical (IHC) staining of the tumor samples was evaluated and scored by two pathologists who were also responsible for generating of the clinicopathological data. The estrogen receptor (ER) and progesterone receptor (PR) were detected using the specific monoclonal antibodies 1D5 and PgR 636 (DAKO), respectively. The cut-off values for the ER and PR statuses were 10% positively stained cells. The HER2 analysis was performed using the HercepTest™ (DAKO CYTOMATION code K5204). When a result of +2 positive was obtained, an *in situ* fluorescent hybridization (FISH) assay was performed to confirm the result. Other clinicopathological data (e.g., tumor size, local recurrence, metastasis and death) are summarized in Table 1.

### RNA extraction and reverse transcription

Total RNA was isolated using the TRIzol® Reagent (Invitrogen) according to the manufacturer's protocol. Reverse transcription reactions were performed using 500 ng of DNA-free RNA, an oligo (dT)_12–18_ primer and Superscript II Reverse Transcriptase (Gibco, BRL). PCR was performed using *CXCR4*-specific primers and *GAPDH-*specific primers as a positive control (Table 2). The PCR was performed in a 20 µl volume containing 1× PCR buffer (Invitrogen), 1.5 mM of MgCl_2_ (Invitrogen), 200 µM dNTPs, 0.3 µM of each primer and 1 U of Platinum T*aq* (Invitrogen). The PCR conditions were as follows: 95°C for 10 min, 94°C for 45 s, the appropriate annealing temperature for 45 s, 72°C for 1 min and a final extension of 72°C for 5 min. PCR products were resolved on 1% agarose gels and stained with ethidium bromide.

**Table 2:** Sequence of the primers used for RT-PCR, nested-PCR and MSP.

Application and specificity	Forward primer (5′-3′)	Reverse primer (5′-3′)	Product size (bp)	Annealing Temperature (°C)
***RT-PCR***				
**CXCR4**	CAGCAGGTAGCAAAGTGA	AGCGTGATGACAAAGAGG	**389**	**58**
**GAPDH**	CTGCACCACCAACTGCTTA	CATGACGGCAGGTCAGGTC	**296**	**63**
***nested-PCR***				
**CXCR4**	AGGAAATGTTTTTGGGAGGTTTTG	TTTTGATTTGAATGTGATTAGGG	**-**	**50, 52, 54**
**CXCR4 nested**	AGTAGGGTTTTTTGGGTTTTTTAAGT	TTGGTTGTTTGATTTTAAAGATTGG	**184**	**52, 54, 56**
**SATR-1**	GTTATATTATTTTTTGTTTTTTTG	ACATTTCCTTATAATATTATTCC	**-**	**48, 50, 52**
**SATR-1 nested**	TATAGTGGTGGTGTATATTTG	CACCTAACCTATAATATTTCTTC	**690**	**52, 54, 56**
***MSP-PCR***				
**CXCR4 – M**	CGCGTATTTTTTCGTTTCG	AATCGCCGCATACGCAGC	**99**	**61**
**CXCR4 – U**	AAGTTGTGTATTTTTTTGTTTTG	ACATACACAACACAAACCTCAC	**110**	**50**

**Abbreviations:** M, specific for methylated condition; U, specific for unmethylated condition.

### 5-aza-2′-deoxycytidine (5-aza-CdR) treatment

The MDA-MB-435 cell line were plated (1×10^6^ cells/ml) and treated for 7 days with 1 µM 5-aza-CdR (Sigma Aldrich, Geisenhein, Germany) or left untreated for an equivalent time. The media was changed daily, and no significant cell death was observed. After treatment for 7 days, total RNA was isolated. The expression of *CXCR4* in breast tumor cells was analyzed using semi-quantitative RT-PCR with *GAPDH* as an internal control. The PCR products were resolved on a 1% agarose gel and stained with ethidium bromide.

### DNA isolation and sodium bisulfite treatment

Genomic DNA was isolated from the MDA-MB-436, MDA-MB-435, MCF7 and PMC-42 breast cancer cell lines or frozen tumor samples using a phenol/chloroform extraction [35]. The DNA was then subjected to sodium bisulfite treatment using an EpiTect® Bisulfite Kit (Qiagen) according to the manufacturer's instructions.

### *CXCR4* CpG island methylation analysis

The DNA region located between positions −173 and +11 from the 5′-flanking region of the *CXCR4* gene, which contained a 184 bp fragment with 19 CpG dinucleotides, was examined. The DNA fragment was amplified from bisulfite-treated DNA of breast tumor cell lines and tumor samples using a nested-PCR amplification protocol. We designed primers using the *Methprimer* program (http://www.urogene.org/Methprimer/index1.html). Briefly, two sets of primers were used for the nested PCR reactions at their appropriate annealing temperatures. The primer sequences are shown in Table 2. The amplified products were purified using a Qiaquick Gel Extraction Kit (Qiagen) and cloned into the pCR2.1 cloning vector (Invitrogen). Eight clones were sequenced for each cell line using the universal or reverse primers. The DNA sequencing reactions were performed using Big Dye Terminator technology on ABI 377 sequencer (Applied Biosystems) according to the manufacturer's instruction. One hundred percent methylation was obtained if a methylated cytosine in the CpG dinucleotide was present in all eight sequenced clones. The methylation percentage for each tumor cell line (global methylation pattern) was calculated by dividing the number of methylated CpG dinucleotides by the total number of CpGs analyzed.

### Methylation-specific PCR (MSP)

After sequencing a 184 bp fragment from the bisulfite treated DNA, we identified the differentially methylated CpG dinucleotides in samples that expressed and did not express the *CXCR4* gene. We designed primers for MSP according to the method described by Herman *et al.* [36]. The genomic DNA (gDNA) from the primary breast tumors was treated with sodium bisulfite and amplified with the *CXCR4* primers specific for the methylated (M) and unmethylated (U) DNA (Table 2).

MSP reactions were performed with 1 µl of bisulfite-modified DNA, 1× PCR buffer (Invitrogen), 1.5 mM of MgCl_2_, 200 µM dNTPs, 0.3 µM of each primer and 1 U of Platinum *Taq* (Invitrogen). The PCR protocol conditions were as follows: 95°C for 10 min; 38 cycles of 94°C for 45 s, the appropriate annealing temperature for 30 s and 72°C for 45 s, followed by a final extension of 72°C for 5 min. The DNA conversion efficiency was confirmed using a nested-PCR reaction with a set of primers for a previously described satellite region [37]. This reaction was used as a control for the bisulfite modification quality. The PCR reaction, nested PCR and temperature conditions are all described in Table 2. The amplification products were separated on 2% agarose gels and stained with ethidium bromide.

### Statistical analyses

The statistical analyses were performed using the SPSS program (version 16.0, SPSS Inc., Chicago, Illinois, USA). Associations between the specific histopathological and clinical parameters were analyzed using a chi-squared test and Fisher's exact test. The survival function was calculated from the time of disease onset to the occurrence of death. The survival data were censored on April 30^th^, 2010, the final date that the survival data were correlated with the death registry. This resulted in a mean survival of 103 months after the onset of the disease. The Kaplan-Meier estimates were presented for the survival functions, and the differences in survival were analyzed using the log rank test. Cox proportional hazards regression analysis was used to estimate the hazards ratio (HR) and 95% confidence intervals (95% CI) for the overall survival and disease-free survival as defined by local recurrence or distant recurrence, whichever occurred first. Statistical significance was assumed for a *p*<0.05.
